# Ground-State Depletion Nanoscopy of Nitrogen-Vacancy Centres in Nanodiamonds

**DOI:** 10.1186/s11671-021-03503-4

**Published:** 2021-03-10

**Authors:** Jelle Storterboom, Martina Barbiero, Stefania Castelletto, Min Gu

**Affiliations:** 1 Optical Sciences Centre, Faculty of Science, Engineering and Technology, Swinburne University of Technology, PO Box 218, Hawthorn, VIC 3122 Australia; 2 Department of Imaging Physics, Delft University of Technology, Delft, The Netherlands; 3 The Institute of Cancer Research, London, UK; 4 School of Engineering RMIT University, Bundoora, Australia; 5 Laboratory for Artificial-Intelligence Nanophotonics, School of Science, RMIT University, Melbourne, VIC Australia; 6 Centre for Artificial-Intelligence Nanophotonics, School of Optical-Electrical and Computer Engineering, The University of Shanghai for Science and Technology, Shanghai, China

**Keywords:** Nanodiamonds, Nitrogen-vacancy centre, Super-resolution microscopy

## Abstract

The negatively charged nitrogen-vacancy (${\text{NV}}^{ - }$) centre in nanodiamonds (NDs) has been recently studied for applications in cellular imaging due to its better photo-stability and biocompatibility if compared to other fluorophores. Super-resolution imaging achieving 20-nm resolution of ${\text{NV}}^{ - }$ in NDs has been proved over the years using sub-diffraction limited imaging approaches such as single molecule stochastic localisation microscopy and stimulated emission depletion microscopy. Here we show the first demonstration of ground-state depletion (GSD) nanoscopy of these centres in NDs using three beams, a probe beam, a depletion beam and a reset beam. The depletion beam at 638 nm forces the ${\text{NV}}^{ - }$ centres to the metastable dark state everywhere but in the local minimum, while a Gaussian beam at 594 nm probes the ${\text{NV}}^{ - }$ centres and a 488-nm reset beam is used to repopulate the excited state. Super-resolution imaging of a single ${\text{NV}}^{ - }$ centre with a full width at half maximum of 36 nm is demonstrated, and two adjacent ${\text{NV}}^{ - }$ centres separated by 72 nm are resolved. GSD microscopy is here applied to ${\text{NV}}^{ - }$ in NDs with a much lower optical power compared to bulk diamond. This work demonstrates the need to control the NDs nitrogen concentration to tailor their application in super-resolution imaging methods and paves the way for studies of ${\text{NV}}^{ - }$ in NDs’ nanoscale interactions.

## Introduction

Nitrogen-vacancy (${\text{NV}}^{ - }$) centre in diamond, consisting of substitutional nitrogen with a neighbouring vacancy, has attracted a wide interest in several scientific and technological fields, among the most notable as a quantum memory in future quantum computers [1], a very sensitive magnetometer [2] with applications in biomedical imaging of living cells [3] and of neurons activity [4], and as an atomic scale probe in various super-resolution imaging methods such as stimulated emission depletion (STED) microscopy and its spin variant, a variant of ground-state depletion (GSD) microscopy [5–7] and single molecule stochastic localisation microscopy (SMLM) with nanometric spin localisation [8]. In particular, methods beating the diffraction limit in fluorescence microscopy represent a paradigm change in today’s biomedical science [9] and ${\text{NV}}^{ - }$ in diamond has played a relevant role in this area as a novel nanoprobe. Due to the diamond inertness, high biocompatibility, robustness and the photo-stability of ${\text{NV}}^{ - }$ photo-luminescence with high quantum yield, it has been widely explored for applications in biomedical science and bio-photonics [10, 11] and in brain microscopy [12] also in its nanostructure form known as nanodiamonds (NDs) [13, 14]. NDs retain similar NV fluorescence properties of the host bulk diamond with the advantages of being more compatible for life science super-resolution imaging applications [15]. However, due to the inhomogeneity in shapes and material nitrogen doping of the currently mass produced fluorescent NDs, inducing variable properties of the NV compared to bulk diamond and often hosting other impurities, super-resolution imaging using NDs is generally more challenging compared to bulk diamond.

Limitations to apply super-resolution methods to NDs compared to better purity bulk diamond are associated with the variability of the photo-physical properties of the ${\text{NV}}^{ - }$ due to inhomogeneity of the NDs nitrogen concentration, charge traps, and other impurities concentration.

Nanoscale imaging of ${\text{NV}}^{ - }$ centres in NDs has been demonstrated with STED microscopy with a depletion beam power of > 650 mW to achieve 20-nm resolution [16, 17] (the maximum resolution in bulk was achieved with of few 3.7 GW/cm^2^ [5]); however, an imaging modality that achieves nanoscale resolution with optical intensities in the order of μW is necessary for as example nanoscale in vivo cellular imaging to reduce photo-toxicity. NDs have the advantage of enabling cellular labelling, which is not possible with the bulk platform and has been used for super-resolution imaging of magnetic field using SMLM [18], which is less photo-toxic than STED or GSD microscopy. Previous super-resolution imaging of ${\text{NV}}^{ - }$ in bulk diamond using GSD was achieved by depleting its ground state using a high intensity beam of 532 nm exciting the centres to the luminescent state; therefore, depletion of the ground state occurs via the excited state [7]. However, this approach also required very high optical intensities to achieve super-resolution (several GW/cm^2^ to attain a resolution < 10 nm) and an imaging reconstruction algorithm to achieve a positive image [19]. Low power super-resolution has been achieved using picosecond pulsed laser inducing the charge conversion of ${\text{NV}}^{ - }$ into its neutral charge state (${\text{NV}}^{0}$ with a zero phonon line at 575 nm) in bulk diamond, known as charge-state conversion (CSD) depletion nanoscopy [20, 21]; however, the depletion beam average power was 1 mW to attain 12-nm resolution and the mechanism appears to be material dependent, that is ultra-low nitrogen concentration (lower than 5 ppb [21]), generally achieved in electronic grade chemical vapour deposition bulk diamond.

An alternative approach to GSD nanoscopy of ${\text{NV}}^{ - }$ centres in bulk diamond used the metastable dark state to deplete the ground state using much lower power via a long-lived metastable state, as originally proposed in Ref. [22] and first demonstrated in Ref. [23] in mammalian cell using organic dye Atto532.

The principle of GSD is the deactivation of the fluorescence of a fluorescent marker through a two-beam approach. The first beam is the excitation or probe beam, which excites the fluorophore to the excited state, and the second beam is the inhibition beam, which switches off the fluorescence. The deactivation of fluorescence is achieved by transiently shelving the population of a fluorophore to a metastable state or a long-lived triplet state. Optical transitions between singlet metastable states and triplet states require a spin flip and are therefore optically suppressed [23].

In Ref. [24], GSD was enabled by continuously pumping the centre from 25 to 200 μW optical power from a CW red laser (638 nm) that brings the ${\text{NV}}^{ - }$ into a non-fluorescent state by populating its long-lived metastable state, thus depleting its ground state, while a blue laser (476 nm) was used to empty the dark metastable state inducing transitions to higher energy states. Here the 638-nm laser is used also to excite the centre even if not efficiently and then to shelve it in its metastable state. ${\text{NV}}^{ - }$ showed a luminescence only when excited with both blue and red lasers. A resolution of 16 nm was achieved with 5 mW of depletion laser power, which is much lower than what used in STED or previous GSD. One possible limitation of this method could be the dependence of the properties of the metastable state from different types of diamond with less or more nitrogen content, or even more in NDs, limiting its applicability. In Ref. [24], the bulk diamond was a type IIa optical grade diamond, which corresponds to a nitrogen concentration of 500 ppm, by far higher than the more recent work on CSD [21], which relies on low nitrogen concentration (< 5 ppb). The statement reported in Ref. [21] that the dark state of Ref. [24] is the neutral charge state of the ${\text{NV}}^{ - }$ suffers from not considering that in high nitrogen concentration the NV^−/0^ charge conversion is suppressed [25] and NV is predominantly in its negative charge state; further, the measured lifetime of the dark state is very long and cannot be attributed to a charge conversion process. In fact, CSD has not yet been proved in commercial fluorescent NDs (derived from high pressure and high temperature, HPHT, diamond) due to their high nitrogen concentration, charge instability due to other defects and photo-physical variable properties due to the lack of material control.

The mechanism to establish GSD nanoscopy has also yet not been reported with ${\text{NV}}^{ - }$ centres in NDs due to the hypothesis that CSD could not occur in NDs.

In this paper, we show that GSD can be performed in NDs, proving the dependence of GSD and CSD methods on the diamond nitrogen concentration and prompting the need of NDs material engineering for specific super-resolution and spin sensing methods.

In this paper, we demonstrate GSD nanoscopy of ${\text{NV}}^{ - }$ centres in NDs using a similar on/off fluorescent switching mechanism achieved by shelving the electrons in the long-lived metastable state using a three-beam approach and using 300 μW to completely deplete the excited state and achiever the maximum resolution. Here, we show that the properties of the ${\text{NV}}^{ - }$ in NDs do not limit the applicability of this method, as previously thought [24]. Further we were able to distinguish two ${\text{NV}}^{ - }$ centres within the same NDs as shown previously using SMLM [26] and STED microscopy [17].

To substantiate that the mechanism of ground-state depletion in our work is due to a dark-lived metastable state rather than CSD, we will summarise the key recent results in relation to the NV^−/0^ charge state conversion. In this regard to photo-induce ionisation, recombination of the NV^−/0^ charge states has been thoroughly studied and found to be excitation wavelength dependent [27, 28], with charge-state switching for red (or blue) laser excitation, with red excitation used to switch the NV^−^ into its neutral charge state. This fast switching occurring at low laser power has been used for implementing charge-state depletion nanoscopy [21] and stochastic optical reconstruction nanoscopy [8] only in bulk diamond grown by chemical vapour deposition, where the nitrogen concentration is well controlled and generally very low due to low-temperature growth. This switching dynamics due to the excitation wavelength based on charge-state conversion of the NV was not observed in NDs [18, 26, 29], where blinking was due to charge traps produced by mechanical damage during fabrication process (generally crashing HPHT high nitrogen concentration microdiamonds) or other effects associated with oxidation [26, 29–31] or proximity with other acceptors impurity [18, 32], and as such not related to NV^−/0^ charge conversion. It has been also demonstrated that NV^−^ charge conversion also strongly depends on the concentration of electron donor impurities in the diamond lattice, namely the negative state conversion to neutral charge state is suppressed for high concentration of donors impurities (Nitrogen) concentration [25]. It has in fact been proposed to use donor and acceptors in diamond to stabilise NV charge state [33]. Most of these studies were conducted in bulk diamond and only a recent work [34] showed that the growth of NDs at low temperature permits a lower nitrogen incorporation, and as such NDs behave as pure bulk diamond with strong charge-state switching for red (or blue) laser excitation, while high-temperature growth incorporating more nitrogen impurities suppresses significantly this charge conversion.

In our NDs, we have not observed NV^−/0^ charge conversion due to laser wavelength excitation, and we attribute the dark state indeed to the metastable state as previously done [24], also in view of the large size of the NDs here used (~ 100 nm), with no blinking due to surface charge states, in addition more importantly to their high nitrogen concentration. The NDs here studied are commercially derived from HPHT microdiamonds with at least inhomogeneous 500 ppm nitrogen concentration. The metastable state in Ref. [24] is also proved by its very long lifetime of 150 s.

${\text{NV}}^{ - }$ centres are generally best excited using 532 or 561 nm as they possess a zero phonon line at 637 nm [35], corresponding to the transition between a triplet ground state and excited state, predominantly a spin conserving transition. Additionally a singlet metastable state exists [36] over which the centre transits via non-radiative decay. The optical transition lifetime of ${\text{NV}}^{ - }$ in NDs is around 22 ns [37], longer compared to the one in bulk diamond of 12 ns [35].

**Fig. 1:**
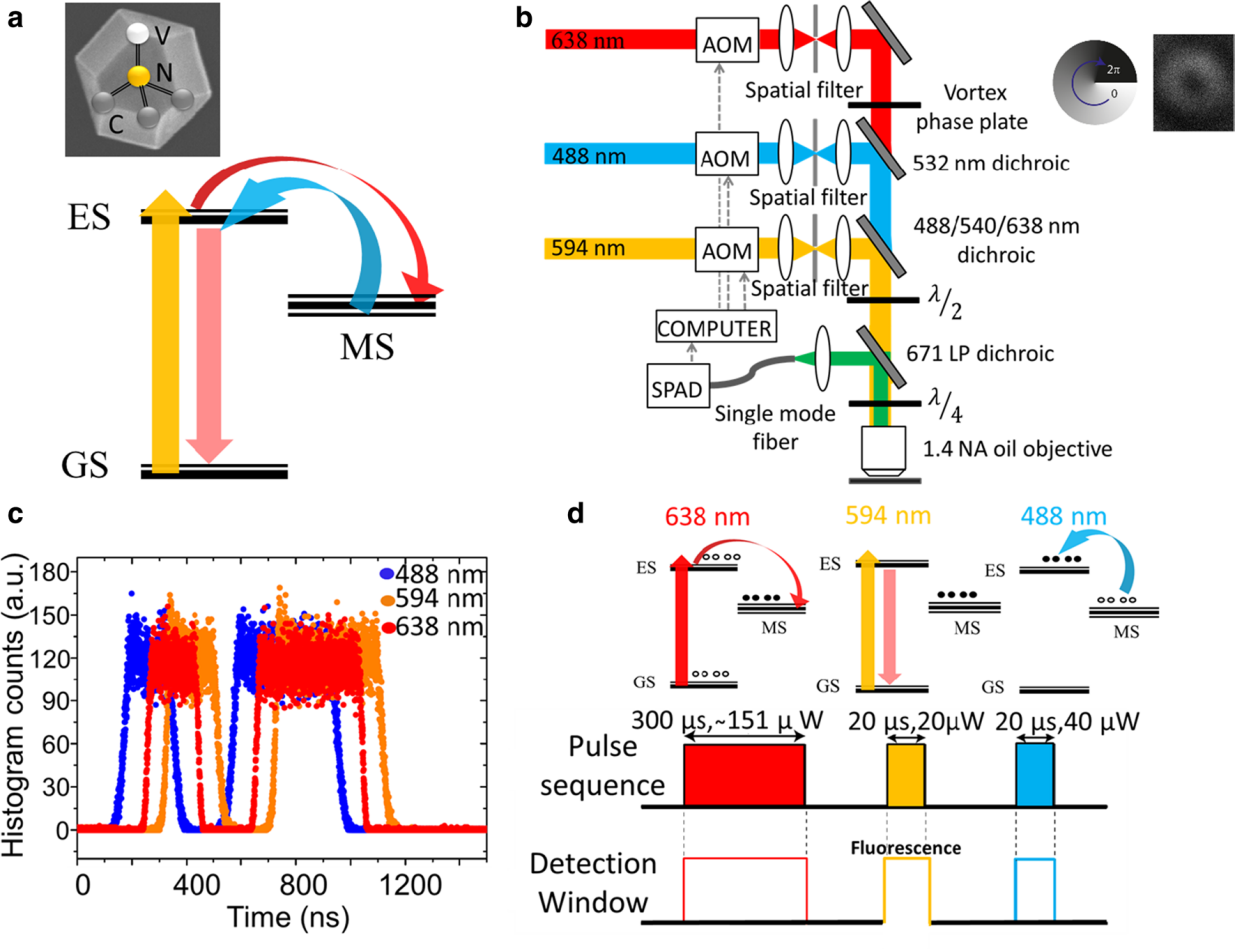
**a** Principle of the GSD in ${\text{NV}}^{ - }$ in NDs using a probe beam at 594 nm (yellow), a depletion beam at 638 nm (red) and a reset beam at 488 nm (blue). GS ground state, ES excited state and MS metastable state of the NV centre. Illustration of an NV centre in a nanodiamond. **b** Schematic representation of the experimental set-up. The system consists of a home-built confocal microscope with three lasers operating at the wavelengths of 488 nm, 594 nm and 638 nm. A vortex phase plate spatially engineers the 638 nm depletion beam into a donut beam to ensure depletion only around the diffraction limited area. **c** Characterisation of acousto-optic modulators by measuring the pulse arrival times of each laser. **d** Schematic of the pulse sequence used in GSD nanoscopy. The detection window is synchronised to the probe beam at 594 nm to collect only relevant fluorescence. The pulse length of the 594 nm probe beam was optimised at 20 μs, as a shorter pulse length would result in longer averaging times and longer pulse lengths would lead to less efficient super-resolution imaging. The 488 nm reset beam was optimised at 20 μs as it is preferable to have it as short as possible to reduce the overall pulse sequence time but still empty the long-lived metastable state effectively

An excitation (probe) beam at 594 nm promotes the transition from the ground state to the excited state, while a depletion beam at 638 nm transiently shelves the electron of ${\text{NV}}^{ - }$ centres from the excited state to the metastable state via non-radiative intersystem crossing (Fig. 1a). The use of 594 nm as probe is justified by its minimal altering of the dark state population if compared to the green (usually 532 or 561 nm) [24]. This method effectively saturates the intersystem crossing and empties the ground state, preventing excitation into the excited state of ${\text{NV}}^{ - }$ centres and subsequent emission of fluorescence, when probed with 594 nm. Finally, a blue laser at 488 nm repopulates the excited state, inhibiting subsequent fast (ns) decay to the ground state [38].

Similarly to STED, the depletion beam is spatially engineered into a donut beam in the transverse plane in order to achieve super-resolution imaging [24]. The resolution *d* of GSD nanoscopy obeys to Eq. [23]:

1 $$d \approx \frac{\lambda }{{[2\left( {NA} \right)\sqrt {1 + I_{{\text{D}}}^{\max } /I_{{\text{s}}} ]} }}$$

with *NA* denoting the numerical aperture of the objective lens, $I_{{\text{s}}}$ the saturation intensity at which half of the fluorescence is depleted and $I_{{\text{D}}}^{\max }$ the maximum intensity value of the peak bordering the zero.

We measured an imaging resolution of 36 nm for a single ${\text{NV}}^{ - }$ centre in ND depending on the depletion beam intensity. Furthermore, two ${\text{NV}}^{ - }$ centres separated by 72 nm have been resolved. Our work is promising for fluorescent nanoscopy within live cells with NDs containing ${\text{NV}}^{ - }$ centres as biomarkers.

## Experimental Methods

For this experiment, a suspension of high-pressure high-temperature (HPHT) NDs nominally 100 nm in size [39, 40], acid-cleaned and diluted in MilliQ solution, was used. A volume of 20 µl NDs solution (1:200 diluted in MilliQ water) was drop-cast on oxygen plasma asher-cleaned borosilicate coverslip and dried in air [37]. The experimental set-up for GSD nanoscopy consisted of a home-built confocal microscope with two continuous wave diode lasers at wavelengths of 488 nm and 638 nm and a continuous wave helium–neon (HeNe) laser at a wavelength of 594 nm. In each of the beam paths, an acousto-optic modulator (AOM) was installed to create optical pulses out of the continuous light beams and synchronise them with respect to each other. After propagating through the AOMs, a spatial filter cleaned up the spatial beam profiles. A secondary lens collimated each beam for further propagation. First the laser beams at the wavelengths of 488 nm and 638 nm were spatially overlapped using a 532-nm long-pass laser-flat dichroic (Semrock- LPD01 532R). A secondary dichroic (Semrock- R405/488/543/638) spatially overlapped all three beams by reflecting the 488 nm and 638 nm wavelengths and transmitting the 594 nm wavelength. The spatial beam profile of the depletion beam at the wavelength of 638 nm was engineered into a donut beam using a phase plate. Further, a quarter wave plate was placed before the objective to ensure circular polarisation of the depletion beam. A high numerical aperture (NA = 1.4) oil immersion objective was used to image the NDs. A dichroic (Semrock- LP02-671RU-25) separated the fluorescence of the ${\text{NV}}^{ - }$ centres from the excitation wavelengths and redirected it to a single-photon Avalanche diode (SPCM-AQRH-14FC) detector via a single-mode fibre acting as a pinhole. The schematic representation of the optical set-up is shown in Fig. 1b.

Gold nanoparticles of the average size of 40 nm were drop-cast onto a glass coverslip and imaged to investigate the spatial profile of the depletion beam. A donut-shaped point spread function with a ring of high intensity and a local minimum in its centre is observed (inset of Fig. 1b).

We first investigated the temporal response of each laser pulse. The AOMs are controlled through LabVIEW using a multichannel pulse generator (PulseBlasterESR-PRO) that controls the individual temporal properties of each laser beam, as well as the timing between the three beams. Due to different optical path lengths for each of the lasers, optical pulses that are generated at the same time have a slightly offset arrival time at the detector. This allows us to have temporal control over all three laser beams at the sub-microsecond timescale.

A rise and fall time of 60 ns was measured for the excitation beams (Fig. 1c). The optical pulses at the wavelengths of 594 nm and 638 nm were arrived with a separation of 85 ns and 155 ns with respect to the pulse at 488 nm wavelength. Each of the laser pulses generates fluorescence that originates from several different sources, including the ${\text{NV}}^{ - }$ centre, other impurities in the ND and defects within the glass coverslip. Only fluorescence generated by the excitation beam is relevant for GSD imaging. The fluorescence from other laser pulses contributes to the noise of the fluorescence image. For that reason, time gating is introduced to eliminate fluorescence generated by laser pulses other than the probe beam. This is achieved through gating the detection with the pulse arrival time of the probe beam, effectively filtering out the other fluorescent sources and increasing the signal-to-noise ratio of the imaging.

**Fig. 2:**
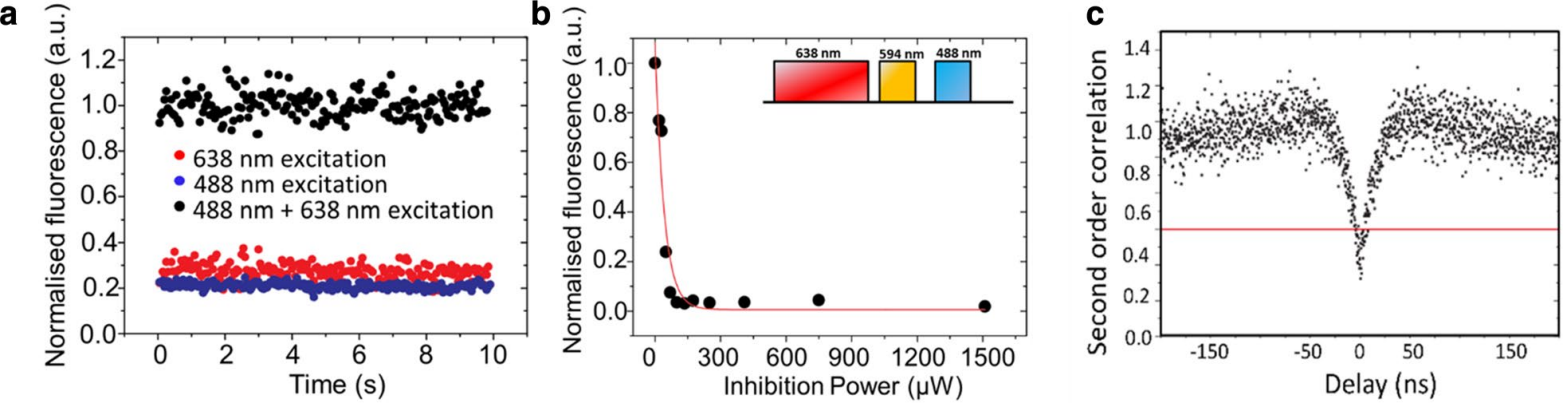
**a** The fluorescence of a single ${\text{NV}}^{ - }$ centre based on 488 nm and 638 nm excitation. The detected fluorescence increases when both lasers simultaneously excite the ${\text{NV}}^{ - }$ centre. **b** Fluorescence dependence on depletion power. An illustration of the pulse sequence is shown in the inset. Fluorescence is detected only when the probe laser at 594 nm is on. **c** Antibunching curve of the ${\text{NV}}^{ - }$ centre showing a second-order correlation function < 0.5 a zero-delay time

A typical pulse sequence for GSD nanoscopy and the detection window is given in Fig. 1d. Switching off the ${\text{NV}}^{ - }$ centres fluorescence was achieved by shelving electrons into the long-lived state with a depletion pulse at the wavelength of 638 nm. Excitation of ${\text{NV}}^{ - }$ centres in the sub-diffraction region was provided by a pulse at the wavelength of 594 nm, while a pulse at 488 nm wavelength reset the ${\text{NV}}^{ - }$ centres back into the excited state. A pulse sequence was repeated 500–1000 times to ensure a high signal-to-noise ratio. The pulse lengths were in the 10–20 μs range for the probe beam (594 nm) and the reset beam (488 nm). The optimum pulse length of the depletion beam was found to be ~ 300 μs (Fig. 2d). The optimisation of the beam pulses duration is based on Ref. [24]; however, a more systematic optimisation should be performed to improve the final resolution. It is expected that the resolution depends on the inhibition beam pulse duration, which is limited by the metastable state lifetime.

## Results and Discussion

The on/off switching mechanism was investigated by exciting an ${\text{NV}}^{ - }$ centre under the laser sources at the wavelengths of 638 nm and 488 nm (Fig. 2a). An increase in the fluorescence emission of ${\text{NV}}^{ - }$ centres was detected only when the two beams were spatially overlapped. This emitting behaviour was understood, assuming that the beam at 638 nm wavelength preferentially shelved electrons in the long-lived state, while the beam at 488 nm allowed spontaneous emission (Fig. 1a). We assumed a lifetime for the long-lived state of ${\text{NV}}^{ - }$ centres shorter (the only measurement for NDs is 33–127 ns from ref.[38], while the singlet state transition is 300 ns [41]) than the metastable state lifetime in bulk diamond (measured 150 s) [24] and much longer than the lifetime of the excited state [37].

A single ${\text{NV}}^{ - }$ centre was selected using anti-bunching (Fig. 2c) measurement to study the power required for the depletion beam to effectively quench fluorescence in the donut shape [42]. A beam at 594 nm wavelength was added to probe whether the single ${\text{NV}}^{ - }$ centre was in its on state or off state. Figure 2b shows the fluorescence dependence of the selected ${\text{NV}}^{ - }$ centre on the intensity of the depletion beam. A rapid decline in fluorescence was observed. At depletion power of 151.2 μW, the ${\text{NV}}^{ - }$ centre was effectively switched off.

Here, for the first time to our knowledge, we demonstrate GSD nanoscopy applied to NDs for super-resolution imaging of single ${\text{NV}}^{ - }$ centres.

To achieve super-resolution via the GSD, all emitters covered by a focused excitation beam are transiently switched off, except at a sub-diffraction-sized region, due to the overlap of the donut-shaped depletion beam transferring the centre to his long-lived dark state. The obtained resolution can be quantified by the profile in the focal plane where the centres are “on” and it scales as per Eq. (1), where $I_{s}$ scales inversely with the lifetime of the states involved and with the inhibition cross section of the optical switch-off transition [43, 44].

**Fig. 3:**
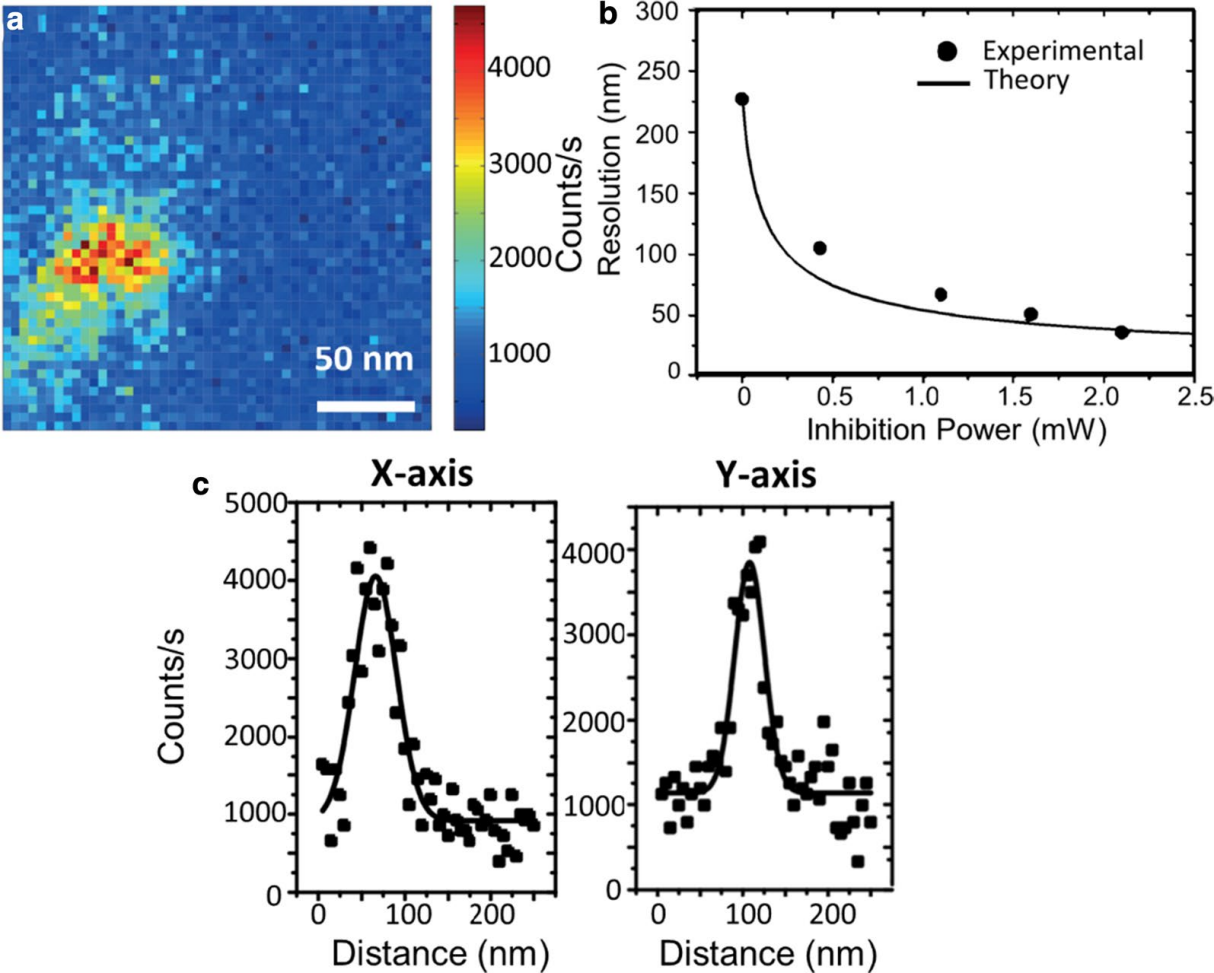
**a** Fluorescence image of a super-resolved ${\text{NV}}^{ - }$ centre based on GSD nanoscopy. Dwell time 10 ms and 2 nm pixel size. **b** Transverse resolution as a function of depletion power. The discrepancy between experiment and theory is most likely due to imperfections in the host material and changes in the local environment of NDs compared to bulk diamond. The theory is based on NV centres in bulk diamond. **c** Image cross sections profile along the x-axis and y-axis with a FWHM resolution of 57 nm and 42 nm, respectively. The solid line represents the best fit

Figure 3a shows a super-resolved fluorescence image of a single ${\text{NV}}^{ - }$ centre in a ND (Fig. 2c). The full width at half maximum (FWHM) is 57 nm in x-direction and 42 nm in y-direction (Fig. 3c).

The relationship between depletion power and resolution is shown in Fig. 3b. The theoretical curve is based on Eq. (1). The additional power required to suppress the ${\text{NV}}^{ - }$ centre fluorescence for the experimental data was attributed to the local environment in the ND host and a shorter metastable state lifetime [38] compared to bulk. It is noted that the maximum resolution of 42 nm is achieved at 2.2 mW, while in Ref. [24] 12-nm resolution was achieved with 16 mW, corresponding to a peak intensity of 12 MW/cm^2^ in for the depletion beam.

**Fig. 4:**
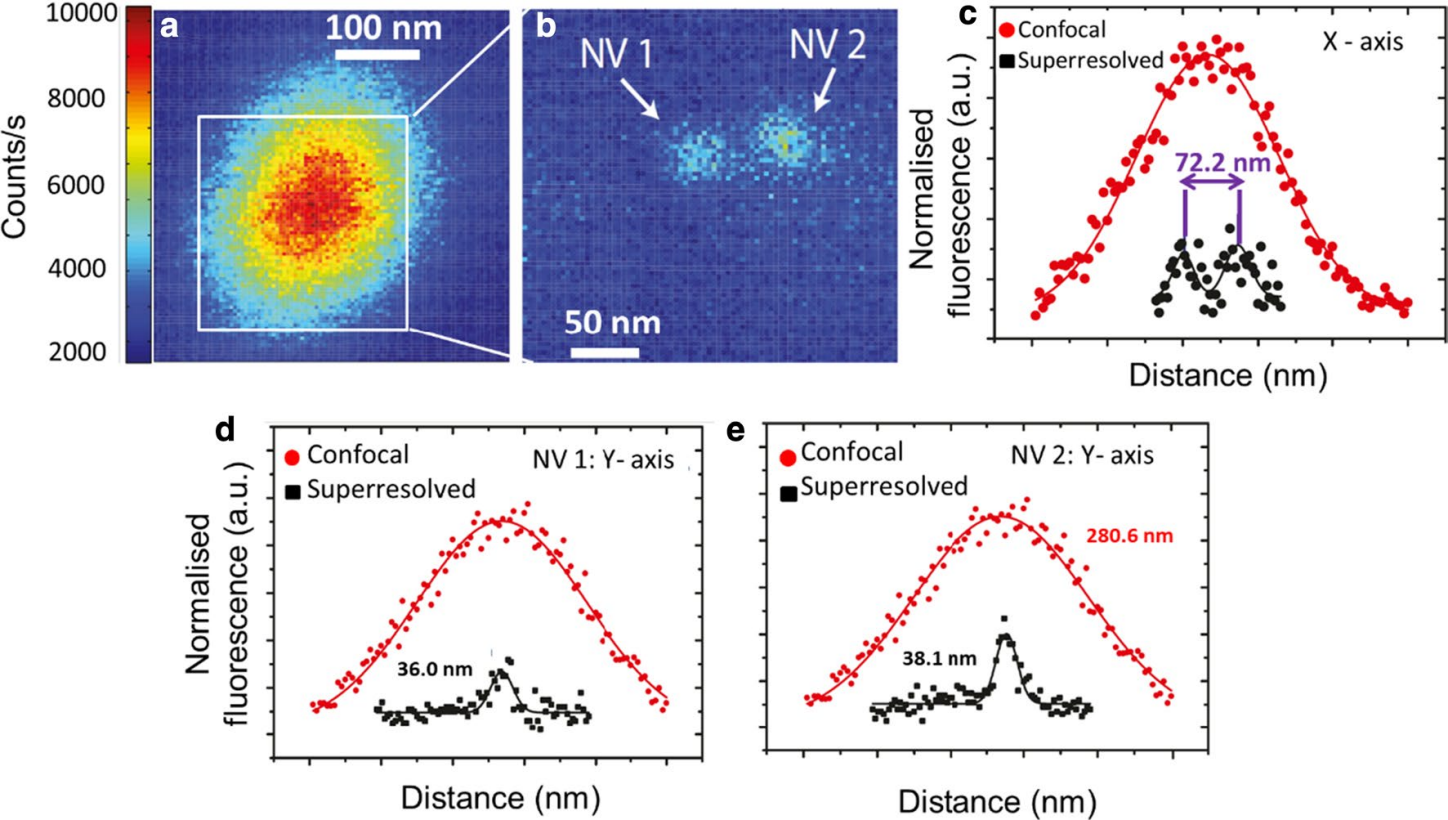
**a** Confocal fluorescence map of 500 × 500 nm (dwell time 2 ms and 1 nm pixel size) and **b** super-resolved image 300 × 300 nm of the white square in (dwell time 24 ms and 1 nm pixel size) **b** of two ${\text{NV}}^{ - }$ centres. The individual confocal spot is super-resolved into two separate dim fluorescence spots. **c** Cross section along the X-axis showing the separation of 72 nm between the two peaks corresponding to the locations of the centre-to-centre distance of the two NV centres. **d** Cross sections along the Y-axis with a measured FWHM resolution of 36 nm and 38 nm, corresponding to ${\text{NV}}^{ - }$ 1 and ${\text{NV}}^{ - }$ 2 in **b**, respectively

Further, we applied GSD nanoscopy to demonstrate nanoscale imaging of two closely spaced ${\text{NV}}^{ - }$ centres. Figure 4a shows a confocal image of ${\text{NV}}^{ - }$ centres. Under GSD nanoscopy, two single ${\text{NV}}^{ - }$ centres are imaged with nanoscale resolution (Fig. 4b). The centre-to-centre distance between the two ${\text{NV}}^{ - }$ centres is 72 nm (Fig. 4c). The FWHM for the two resolved ${\text{NV}}^{ - }$ centres in the *y*-direction is 36 nm (Fig. 4d) and 38 nm, respectively (Fig. 4e). We attributed the misalignment between the major axis of the ND and the resolved ${\text{NV}}^{ - }$ centres to a mechanical drift during the confocal image scanning.

## Conclusions

In this work, we have implemented GSD nanoscopy using the metastable state of ${\text{NV}}^{ - }$ centre in nanodiamonds, showing super-resolved imaging of ${\text{NV}}^{ - }$ centres. A single ${\text{NV}}^{ - }$ centre with a FWHM of 36 nm is resolved. The scaling of the resolution with respect to the intensity of the depletion beam is also shown, and the maximum super-resolution is achieved with 2.2 mW of depletion power. Furthermore, two ${\text{NV}}^{ - }$ centres separated by 72 nm were resolved. This result opens up the possibility of investigating dipolar coupling between closely spaced ${\text{NV}}^{ - }$ centres [45], the ability for high spatial resolution quantum sensing based on the ${\text{NV}}^{ - }$ spin properties [6, 18, 46–48] as well as other multifunctional sensing applications based on bulk diamond [49] extended to NDs [50].

In this paper, we have also shown that the nitrogen concentration in diamond is at the basis of GSD and CSD mechanisms for super-resolution, which are achieved in high and low nitrogen-doped diamond, respectively. As such, engineered NDs for the specific method should follow. It is understood that CSD can be more easily applied to spin sensing due to low nitrogen concentration; however, the NV^0^ charge state would limit the quantum sensing properties of CSD, while stabilising the charge state of ${\text{NV}}^{ - }$ using other donors than nitrogen could bring a much higher sensitivity of the GSD applied to spin quantum sensing.

GSD nanoscopy of ${\text{NV}}^{ - }$ centres in NDs uses low power (~ 300 μW) for the optical intensities compared to bulk diamond, and it is more suitable for biological samples. The GSD resolution can be improved by determining the optimal nitrogen doping [34], by studying the effect of surface passivation and other impurities on the ${\text{NV}}^{ - }$ in NDs metastable state lifetime and by engineering NDs with less photo-luminescent impurities.

In regard to spin sensing, the high nitrogen concentration in currently commercial NDs derived from HPHT and high nitrogen content microdiamonds is limiting the spin sensitivity as per reduced optically detected magnetic resonance contrast [34]. As such, other dopants should be considered for stabilising the ${\text{NV}}^{ - }$ charge state without introducing decoherence [50, 51].

Finally, combining this method with the microwave excitation [18, 52–54], it could provide an alternative approach for super-resolved optical magnetic imaging in life science if the current NDs material properties could be better engineered for this application.

## Data Availability

The datasets used and/or analysed during the current study are available from the corresponding author on reasonable request.

## Abbreviations

NDs: Nanodiamonds
NV: Nitrogen-vacancy
STED: Stimulated emission depletion
GSD: Ground-state depletion
CSD: Charge-state depletion
FWHM: Full width at half maximum
HPHT: High pressure and high temperature
AOM: Acousto-optic modulator
